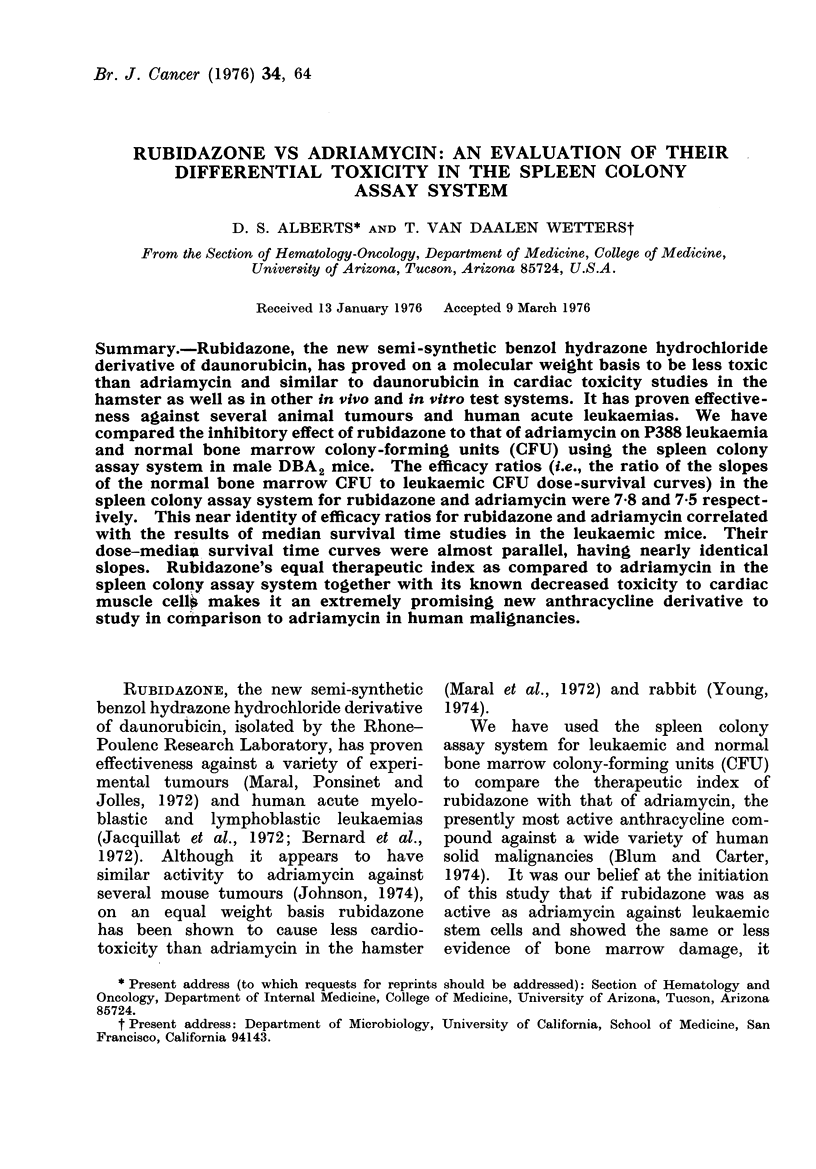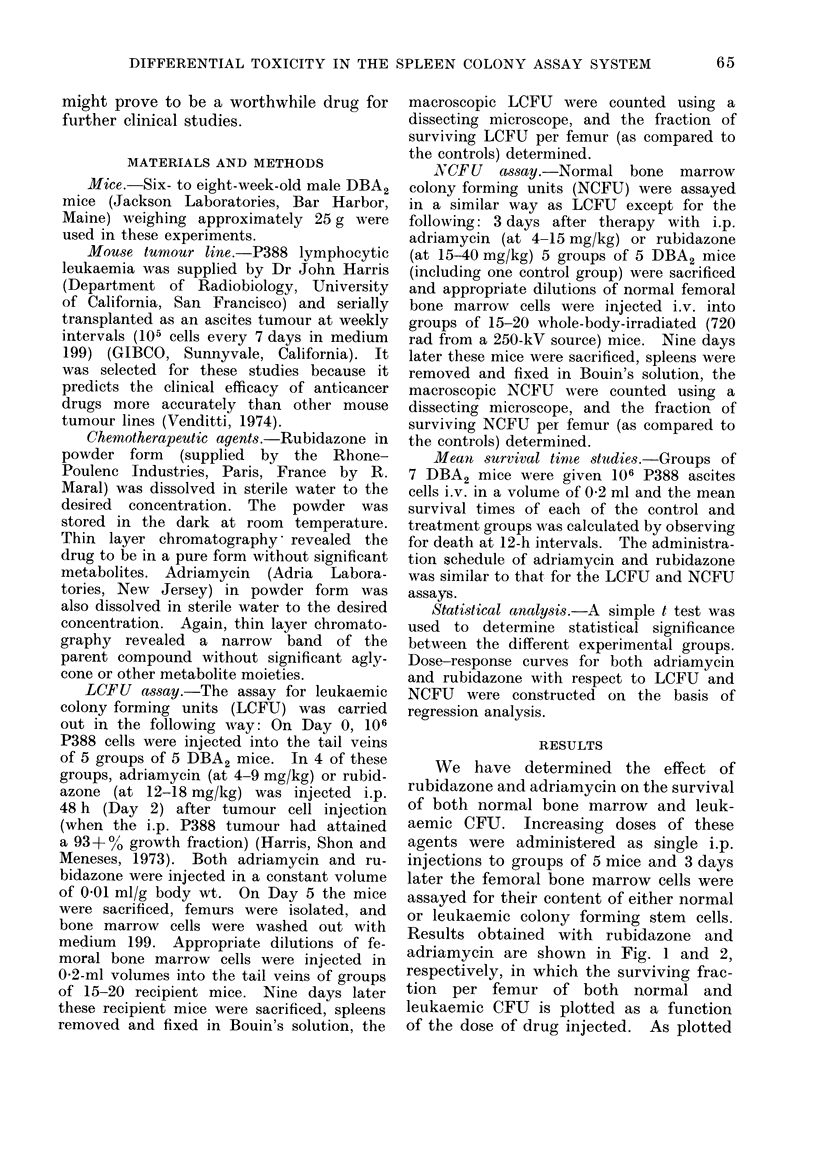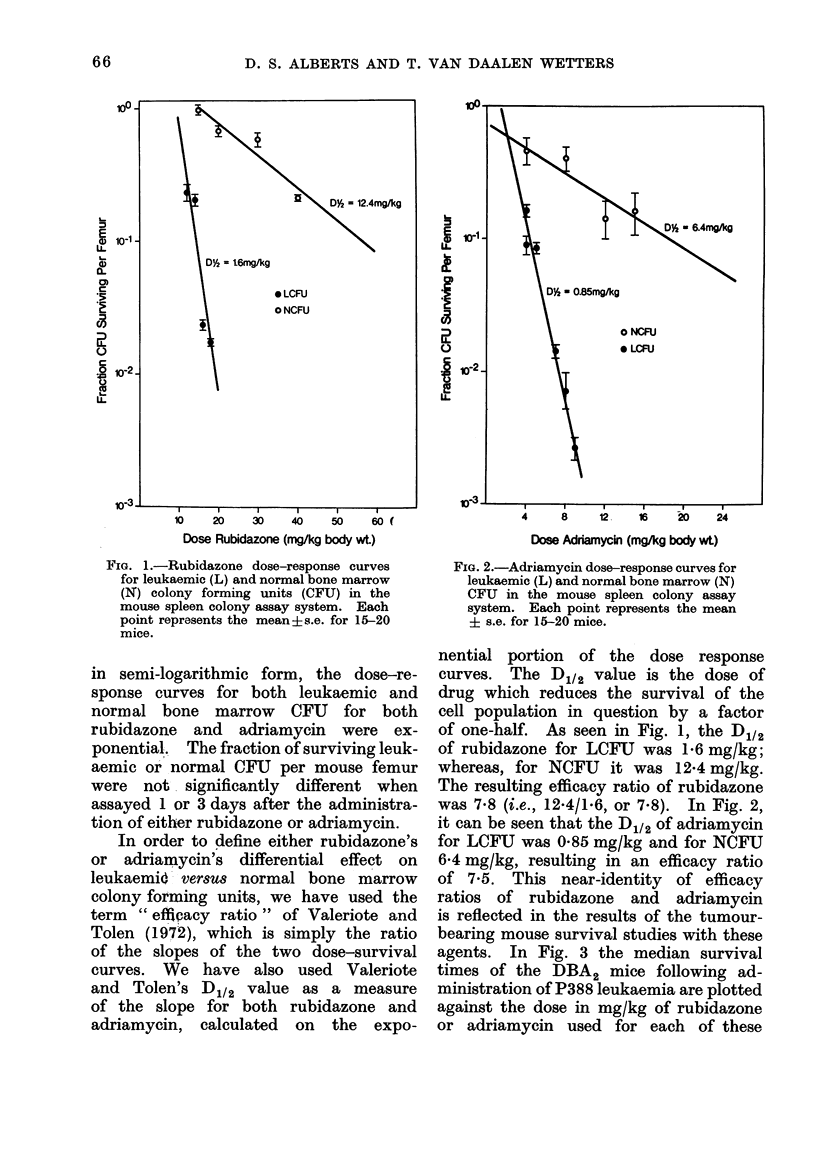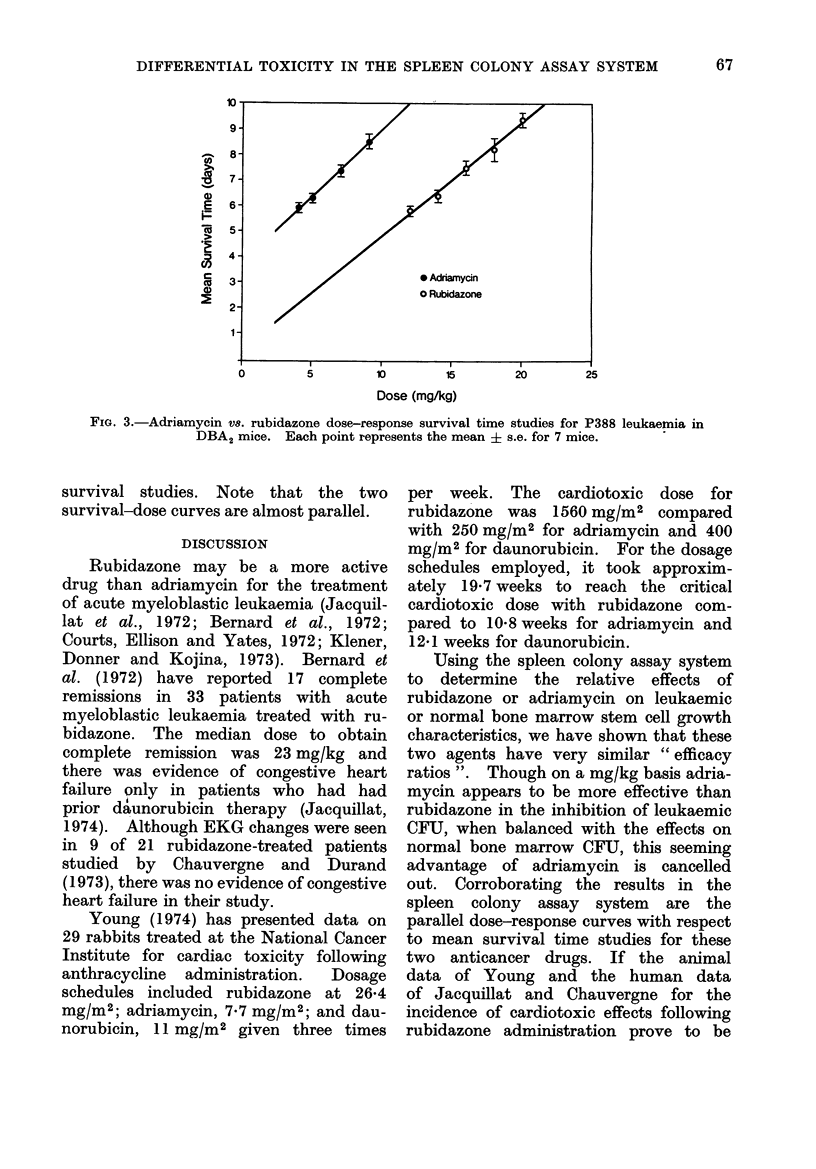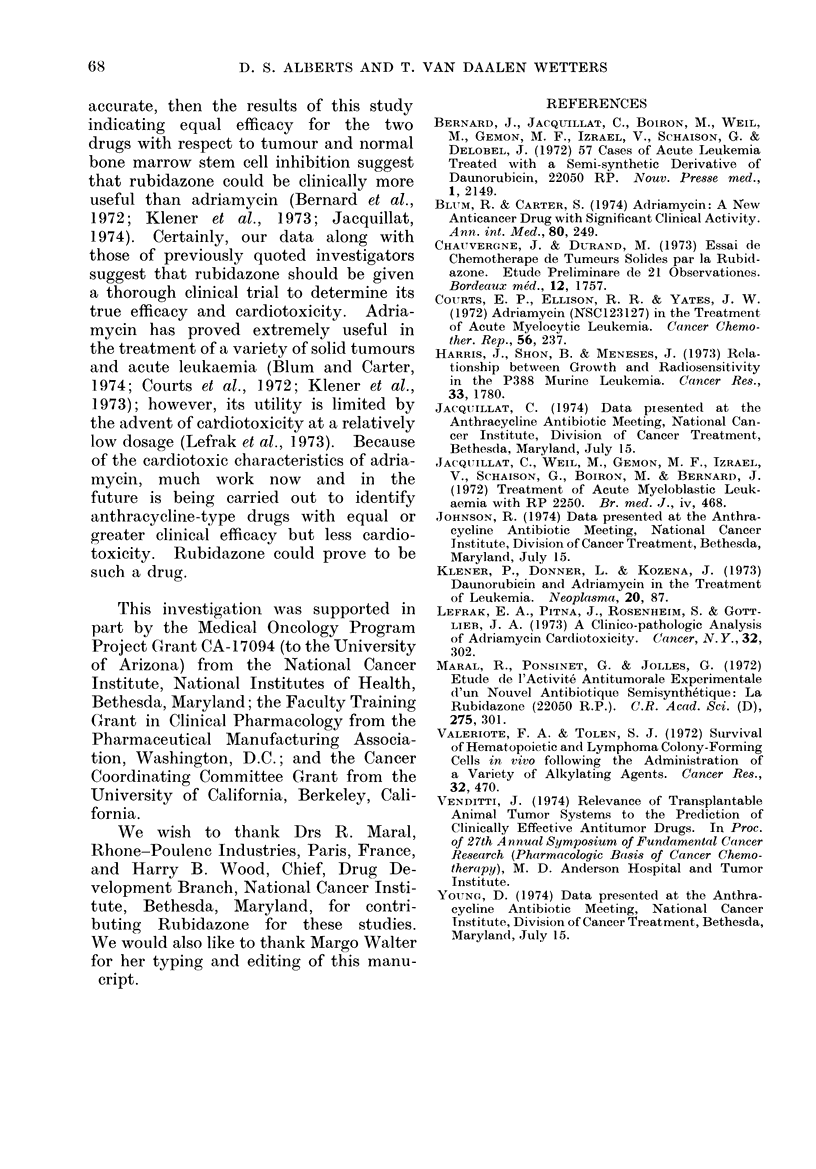# Rubidazone vs adriamycin: an evaluation of their differential toxicity in the spleen colony assay system.

**DOI:** 10.1038/bjc.1976.123

**Published:** 1976-07

**Authors:** D. S. Alberts, T. Van Daalen Wetters

## Abstract

Rubidazone, the new semi-synthetic benzol hydrazone hydrochloride derivative of dauorubicin, has proved on a molecular weight basis to be less toxic than adriamycin and similar to daunorubicin in cardiac toxicity studies in the hamster as well as in other in vivo and in vitro test systems. It has proven effectiveness against several animal tumours and human acute leukaemias. We have compared the inhibitory effect of rubidazone to that of adriamycin on P388 leukaemia and normal bone marrow colony-forming units (CFU) using the spleen colony assay system in male DBA2 mice. The efficacy ratios (i.e., the ratio of the slopes of the normal bone marrow CFU to leukaemic CFU dose-survival curves) in the spleen colony assay system for rubidazone and adriamycin were 7-8 and 7-5 respectively. This near identity of efficacy ratios fro rubidazone and adriamycin correlated with the results of median survival time studies in the leukaemic mice. Their dose-median survival time curves were almost parallel, having nearly identical slopes. Rubidazone's equal therapeutic index as compared to adriamycin in the spleen colony assay system together with its known decreased toxicity to cardiac muscle cells makes it an extremely promising new anthracycline derivative to study in comparison to adriamycin in human malignancies.


Br. J. Cancer (1976) 34, 64

### RUBIDAZONE VS ADRIAMYCIN: AN EVALUATION OF THEIR DIFFERENTIAL TOXICITY IN THE SPLEEN COLONY ASSAY SYSTEM

D. S. ALBERTS* AND T. VAN DAALEN WETTERS†

*From the Section of Hematology-Oncology, Department of Medicine, College of Medicine, University of Arizona, Tucson, Arizona 85724, U.S.A.*

Received 13 January 1976 Accepted 9 March 1976

**Summary.—Rubidazone, the new semi-synthetic benzol hydrazone hydrochloride derivative of daunorubicin, has proved on a molecular weight basis to be less toxic than adriamycin and similar to daunorubicin in cardiac toxicity studies in the hamster as well as in other *in vivo* and *in vitro* test systems. It has proven effectiveness against several animal tumours and human acute leukaemias. We have compared the inhibitory effect of rubidazone to that of adriamycin on P388 leukaemia and normal bone marrow colony-forming units (CFU) using the spleen colony assay system in male $DBA_2$ mice. The efficacy ratios (*i.e.*, the ratio of the slopes of the normal bone marrow CFU to leukaemic CFU dose-survival curves) in the spleen colony assay system for rubidazone and adriamycin were 7·8 and 7·5 respectively. This near identity of efficacy ratios for rubidazone and adriamycin correlated with the results of median survival time studies in the leukaemic mice. Their dose–median survival time curves were almost parallel, having nearly identical slopes. Rubidazone's equal therapeutic index as compared to adriamycin in the spleen colony assay system together with its known decreased toxicity to cardiac muscle cells makes it an extremely promising new anthracycline derivative to study in comparison to adriamycin in human malignancies.**

RUBIDAZONE, the new semi-synthetic benzol hydrazone hydrochloride derivative of daunorubicin, isolated by the Rhone–Poulenc Research Laboratory, has proven effectiveness against a variety of experimental tumours (Maral, Ponsinet and Jolles, 1972) and human acute myeloblastic and lymphoblastic leukaemias (Jacquillat *et al.*, 1972; Bernard *et al.*, 1972). Although it appears to have similar activity to adriamycin against several mouse tumours (Johnson, 1974), on an equal weight basis rubidazone has been shown to cause less cardiotoxicity than adriamycin in the hamster (Maral *et al.*, 1972) and rabbit (Young, 1974).

We have used the spleen colony assay system for leukaemic and normal bone marrow colony-forming units (CFU) to compare the therapeutic index of rubidazone with that of adriamycin, the presently most active anthracycline compound against a wide variety of human solid malignancies (Blum and Carter, 1974). It was our belief at the initiation of this study that if rubidazone was as active as adriamycin against leukaemic stem cells and showed the same or less evidence of bone marrow damage, it

* Present address (to which requests for reprints should be addressed): Section of Hematology and Oncology, Department of Internal Medicine, College of Medicine, University of Arizona, Tucson, Arizona 85724.

† Present address: Department of Microbiology, University of California, School of Medicine, San Francisco, California 94143.

might prove to be a worthwhile drug for further clinical studies.

### MATERIALS AND METHODS

*Mice*.—Six- to eight-week-old male $DBA_2$ mice (Jackson Laboratories, Bar Harbor, Maine) weighing approximately 25 g were used in these experiments.

*Mouse tumour line*.—P388 lymphocytic leukaemia was supplied by Dr John Harris (Department of Radiobiology, University of California, San Francisco) and serially transplanted as an ascites tumour at weekly intervals ($10^5$ cells every 7 days in medium 199) (GIBCO, Sunnyvale, California). It was selected for these studies because it predicts the clinical efficacy of anticancer drugs more accurately than other mouse tumour lines (Venditti, 1974).

*Chemotherapeutic agents*.—Rubidazone in powder form (supplied by the Rhone–Poulenc Industries, Paris, France by R. Maral) was dissolved in sterile water to the desired concentration. The powder was stored in the dark at room temperature. Thin layer chromatography revealed the drug to be in a pure form without significant metabolites. Adriamycin (Adria Laboratories, New Jersey) in powder form was also dissolved in sterile water to the desired concentration. Again, thin layer chromatography revealed a narrow band of the parent compound without significant aglycone or other metabolite moieties.

*LCFU assay*.—The assay for leukaemic colony forming units (LCFU) was carried out in the following way: On Day 0, $10^6$ P388 cells were injected into the tail veins of 5 groups of 5 $DBA_2$ mice. In 4 of these groups, adriamycin (at 4–9 mg/kg) or rubidazone (at 12–18 mg/kg) was injected i.p. 48 h (Day 2) after tumour cell injection (when the i.p. P388 tumour had attained a 93+% growth fraction) (Harris, Shon and Meneses, 1973). Both adriamycin and rubidazone were injected in a constant volume of 0·01 ml/g body wt. On Day 5 the mice were sacrificed, femurs were isolated, and bone marrow cells were washed out with medium 199. Appropriate dilutions of femoral bone marrow cells were injected in 0·2-ml volumes into the tail veins of groups of 15–20 recipient mice. Nine days later these recipient mice were sacrificed, spleens removed and fixed in Bouin's solution, the macroscopic LCFU were counted using a dissecting microscope, and the fraction of surviving LCFU per femur (as compared to the controls) determined.

*NCFU assay*.—Normal bone marrow colony forming units (NCFU) were assayed in a similar way as LCFU except for the following: 3 days after therapy with i.p. adriamycin (at 4–15 mg/kg) or rubidazone (at 15–40 mg/kg) 5 groups of 5 $DBA_2$ mice (including one control group) were sacrificed and appropriate dilutions of normal femoral bone marrow cells were injected i.v. into groups of 15–20 whole-body-irradiated (720 rad from a 250-kV source) mice. Nine days later these mice were sacrificed, spleens were removed and fixed in Bouin's solution, the macroscopic NCFU were counted using a dissecting microscope, and the fraction of surviving NCFU per femur (as compared to the controls) determined.

*Mean survival time studies*.—Groups of 7 $DBA_2$ mice were given $10^6$ P388 ascites cells i.v. in a volume of 0·2 ml and the mean survival times of each of the control and treatment groups was calculated by observing for death at 12-h intervals. The administration schedule of adriamycin and rubidazone was similar to that for the LCFU and NCFU assays.

*Statistical analysis*.—A simple $t$ test was used to determine statistical significance between the different experimental groups. Dose–response curves for both adriamycin and rubidazone with respect to LCFU and NCFU were constructed on the basis of regression analysis.

### RESULTS

We have determined the effect of rubidazone and adriamycin on the survival of both normal bone marrow and leukaemic CFU. Increasing doses of these agents were administered as single i.p. injections to groups of 5 mice and 3 days later the femoral bone marrow cells were assayed for their content of either normal or leukaemic colony forming stem cells. Results obtained with rubidazone and adriamycin are shown in Fig. 1 and 2, respectively, in which the surviving fraction per femur of both normal and leukaemic CFU is plotted as a function of the dose of drug injected. As plotted

FIG. 1.—Rubidazone dose–response curves for leukaemic (L) and normal bone marrow (N) colony forming units (CFU) in the mouse spleen colony assay system. Each point represents the mean ± s.e. for 15–20 mice.

FIG. 2.—Adriamycin dose–response curves for leukaemic (L) and normal bone marrow (N) CFU in the mouse spleen colony assay system. Each point represents the mean ± s.e. for 15–20 mice.

in semi-logarithmic form, the dose–response curves for both leukaemic and normal bone marrow CFU for both rubidazone and adriamycin were exponential. The fraction of surviving leukaemic or normal CFU per mouse femur were not significantly different when assayed 1 or 3 days after the administration of either rubidazone or adriamycin.

In order to define either rubidazone's or adriamycin's differential effect on leukaemic *versus* normal bone marrow colony forming units, we have used the term " efficacy ratio " of Valeriote and Tolen (1972), which is simply the ratio of the slopes of the two dose–survival curves. We have also used Valeriote and Tolen's $D_{1/2}$ value as a measure of the slope for both rubidazone and adriamycin, calculated on the exponential portion of the dose response curves. The $D_{1/2}$ value is the dose of drug which reduces the survival of the cell population in question by a factor of one-half. As seen in Fig. 1, the $D_{1/2}$ of rubidazone for LCFU was 1·6 mg/kg; whereas, for NCFU it was 12·4 mg/kg. The resulting efficacy ratio of rubidazone was 7·8 (*i.e.*, 12·4/1·6, or 7·8). In Fig. 2, it can be seen that the $D_{1/2}$ of adriamycin for LCFU was 0·85 mg/kg and for NCFU 6·4 mg/kg, resulting in an efficacy ratio of 7·5. This near-identity of efficacy ratios of rubidazone and adriamycin is reflected in the results of the tumour-bearing mouse survival studies with these agents. In Fig. 3 the median survival times of the $DBA_2$ mice following administration of P388 leukaemia are plotted against the dose in mg/kg of rubidazone or adriamycin used for each of these

FIG. 3.—Adriamycin *vs.* rubidazone dose–response survival time studies for P388 leukaemia in $DBA_2$ mice. Each point represents the mean ± s.e. for 7 mice.

survival studies. Note that the two survival–dose curves are almost parallel.

### DISCUSSION

Rubidazone may be a more active drug than adriamycin for the treatment of acute myeloblastic leukaemia (Jacquillat *et al.*, 1972; Bernard *et al.*, 1972; Courts, Ellison and Yates, 1972; Klener, Donner and Kojina, 1973). Bernard *et al.* (1972) have reported 17 complete remissions in 33 patients with acute myeloblastic leukaemia treated with rubidazone. The median dose to obtain complete remission was 23 mg/kg and there was evidence of congestive heart failure only in patients who had had prior daunorubicin therapy (Jacquillat, 1974). Although EKG changes were seen in 9 of 21 rubidazone-treated patients studied by Chauvergne and Durand (1973), there was no evidence of congestive heart failure in their study.

Young (1974) has presented data on 29 rabbits treated at the National Cancer Institute for cardiac toxicity following anthracycline administration. Dosage schedules included rubidazone at 26·4 mg/m$^2$; adriamycin, 7·7 mg/m$^2$; and daunorubicin, 11 mg/m$^2$ given three times per week. The cardiotoxic dose for rubidazone was 1560 mg/m$^2$ compared with 250 mg/m$^2$ for adriamycin and 400 mg/m$^2$ for daunorubicin. For the dosage schedules employed, it took approximately 19·7 weeks to reach the critical cardiotoxic dose with rubidazone compared to 10·8 weeks for adriamycin and 12·1 weeks for daunorubicin.

Using the spleen colony assay system to determine the relative effects of rubidazone or adriamycin on leukaemic or normal bone marrow stem cell growth characteristics, we have shown that these two agents have very similar " efficacy ratios ". Though on a mg/kg basis adriamycin appears to be more effective than rubidazone in the inhibition of leukaemic CFU, when balanced with the effects on normal bone marrow CFU, this seeming advantage of adriamycin is cancelled out. Corroborating the results in the spleen colony assay system are the parallel dose–response curves with respect to mean survival time studies for these two anticancer drugs. If the animal data of Young and the human data of Jacquillat and Chauvergne for the incidence of cardiotoxic effects following rubidazone administration prove to be accurate, then the results of this study indicating equal efficacy for the two drugs with respect to tumour and normal bone marrow stem cell inhibition suggest that rubidazone could be clinically more useful than adriamycin (Bernard *et al.*, 1972; Klener *et al.*, 1973; Jacquillat, 1974). Certainly, our data along with those of previously quoted investigators suggest that rubidazone should be given a thorough clinical trial to determine its true efficacy and cardiotoxicity. Adriamycin has proved extremely useful in the treatment of a variety of solid tumours and acute leukaemia (Blum and Carter, 1974; Courts *et al.*, 1972; Klener *et al.*, 1973); however, its utility is limited by the advent of cardiotoxicity at a relatively low dosage (Lefrak *et al.*, 1973). Because of the cardiotoxic characteristics of adriamycin, much work now and in the future is being carried out to identify anthracycline-type drugs with equal or greater clinical efficacy but less cardiotoxicity. Rubidazone could prove to be such a drug.

This investigation was supported in part by the Medical Oncology Program Project Grant CA-17094 (to the University of Arizona) from the National Cancer Institute, National Institutes of Health, Bethesda, Maryland; the Faculty Training Grant in Clinical Pharmacology from the Pharmaceutical Manufacturing Association, Washington, D.C.; and the Cancer Coordinating Committee Grant from the University of California, Berkeley, California.

We wish to thank Drs R. Maral, Rhone–Poulenc Industries, Paris, France, and Harry B. Wood, Chief, Drug Development Branch, National Cancer Institute, Bethesda, Maryland, for contributing Rubidazone for these studies. We would also like to thank Margo Walter for her typing and editing of this manucript.

REFERENCES

BERNARD, J., JACQUILLAT, C., BOIRON, M., WEIL, M., GEMON, M. F., IZRAEL, V., SCHAISON, G. & DELOBEL, J. (1972) 57 Cases of Acute Leukemia Treated with a Semi-synthetic Derivative of Daunorubicin, 22050 RP. *Nouv. Presse med.*, **1**, 2149.

BLUM, R. & CARTER, S. (1974) Adriamycin: A New Anticancer Drug with Significant Clinical Activity. *Ann. int. Med.*, **80**, 249.

CHAUVERGNE, J. & DURAND, M. (1973) Essai de Chemotherape de Tumeurs Solides par la Rubidazone. Etude Preliminare de 21 Observationes. *Bordeaux méd.*, **12**, 1757.

COURTS, E. P., ELLISON, R. R. & YATES, J. W. (1972) Adriamycin (NSC123127) in the Treatment of Acute Myelocytic Leukemia. *Cancer Chemother. Rep.*, **56**, 237.

HARRIS, J., SHON, B. & MENESES, J. (1973) Relationship between Growth and Radiosensitivity in the P388 Murine Leukemia. *Cancer Res.*, **33**, 1780.

JACQUILLAT, C. (1974) Data presented at the Anthracycline Antibiotic Meeting, National Cancer Institute, Division of Cancer Treatment, Bethesda, Maryland, July 15.

JACQUILLAT, C., WEIL, M., GEMON, M. F., IZRAEL, V., SCHAISON, G., BOIRON, M. & BERNARD, J. (1972) Treatment of Acute Myeloblastic Leukaemia with RP 2250. *Br. med. J.*, iv, 468.

JOHNSON, R. (1974) Data presented at the Anthracycline Antibiotic Meeting, National Cancer Institute, Division of Cancer Treatment, Bethesda, Maryland, July 15.

KLENER, P., DONNER, L. & KOZENA, J. (1973) Daunorubicin and Adriamycin in the Treatment of Leukemia. *Neoplasma*, **20**, 87.

LEFRAK, E. A., PITNA, J., ROSENHEIM, S. & GOTTLIEB, J. A. (1973) A Clinico-pathologic Analysis of Adriamycin Cardiotoxicity. *Cancer, N.Y.*, **32**, 302.

MARAL, R., PONSINET, G. & JOLLES, G. (1972) Etude de l'Activité Antitumorale Experimentale d'un Nouvel Antibiotique Semisynthétique: La Rubidazone (22050 R.P.). *C.R. Acad. Sci.* (D), **275**, 301.

VALERIOTE, F. A. & TOLEN, S. J. (1972) Survival of Hematopoietic and Lymphoma Colony-Forming Cells *in vivo* following the Administration of a Variety of Alkylating Agents. *Cancer Res.*, **32**, 470.

VENDITTI, J. (1974) Relevance of Transplantable Animal Tumor Systems to the Prediction of Clinically Effective Antitumor Drugs. In *Proc. of 27th Annual Symposium of Fundamental Cancer Research* (*Pharmacologic Basis of Cancer Chemotherapy*), M. D. Anderson Hospital and Tumor Institute.

YOUNG, D. (1974) Data presented at the Anthracycline Antibiotic Meeting, National Cancer Institute, Division of Cancer Treatment, Bethesda, Maryland, July 15.